# The application of acupuncture in the management of neuropathic orofacial pain: a narrative literature review

**DOI:** 10.22514/jofph.2025.044

**Published:** 2025-09-12

**Authors:** Dan Pan, Yuxiang Pan, Xiao Wang, Hui Wang, Yunyun Lyu, Pan Yang

**Affiliations:** ^1^Department of Rehabilitation Medicine, First Affiliated Hospital of Gannan Medical University, 341000 Ganzhou, Jiangxi, China; ^2^Department of Rehabilitation Medicine, Ganzhou Municipal Hospital, 341000 Ganzhou, Jiangxi, China; ^3^Department of Rehabilitation Medicine, Gannan Healthcare Vocational College, 341000 Ganzhou, Jiangxi, China; ^4^Department of Rehabilitation Sciences, Hong Kong Polytechnic University, 999077 Hong Kong; ^5^Pharmaceutical Compounding Center, Ganzhou People’s Hospital, 341000 Ganzhou, Jiangxi, China; ^6^Department of Dentistry, University of Pécs Medical School, 7624 Pécs, BA, Hungary

**Keywords:** Acupuncture, Neuropathic orofacial pain, Pain management, Neurology, Trigeminal neuralgia, Burning mouth syndrome, Temporomandibular disorders, Quality of life

## Abstract

Neuropathic orofacial pain (NOP), which includes conditions such as trigeminal neuralgia, burning mouth syndrome, and temporomandibular disorders, poses a significant clinical challenge. This difficulty arises primarily from its diverse pathophysiological mechanisms and the limited efficacy of conventional treatments. This review highlights recent progress in understanding the mechanisms underlying NOP while also evaluating the potential of Traditional Chinese Medicine (TCM), with a particular focus on acupuncture, as an integrative therapeutic approach. Emerging clinical studies suggest that acupuncture holds significant potential in alleviating NOP symptoms. Acupuncture effectively manages NOP by combining local (*e.g.*, TE21 (Erheliao), ST7 (Xiaguan), SI18 (Quanliao)) and distal (*e.g.*, LI4 (Hegu), PC6 (Neiguan)) acupoints. Local points relieve pain and improve circulation, while distal points enhance systemic neuromodulation. This integrative approach optimizes pain regulation and neuroinflammatory control, making acupuncture a promising NOP treatment. Various acupuncture techniques and point stimulation methods, whether used independently or as complementary therapies, have demonstrated both safety and efficacy, ranging from traditional practices to more modern innovations. However, the precise mechanisms by which acupuncture exerts its effects in managing NOP remain unclear and warrant further exploration. While interest in TCM and acupuncture for musculoskeletal pain has grown considerably, the evidence specifically addressing NOP remains limited and fragmented. Currently, there are no established protocols or clear clinical guidelines for the use of acupuncture in the treatment of NOP. To bridge this gap, future research should adopt a multidisciplinary approach and implement rigorous experimental designs to deepen our understanding of acupuncture’s underlying mechanisms. Such efforts could provide the foundation for developing standardized protocols, ultimately enhancing acupuncture’s utility as an effective therapeutic option for individuals coping with NOP.

## 1. Introduction

Neuropathic orofacial pain (NOP) is a complex and multifaceted category of chronic pain syndromes that arise from dysfunctions or injuries in the peripheral or central nervous system (CNS) [1, 2, 3]. These conditions affect the oral, facial, cranial, and cervical regions and can be severe enough to significantly impair a person’s quality of life (QoL). Several common types of NOP include burning mouth syndrome (BMS) [4], trigeminal neuralgia (TN) [5], postherpetic neuralgia (PHN) [6] and temporomandibular disorders (TMD) [6]. The pathophysiology of NOP is intricate and involves mechanisms such as peripheral sensitization, central sensitization, neuroinflammation and maladaptive neuroplasticity [7]. These mechanisms contribute to the main symptoms, including spontaneous pain, hyperalgesia and allodynia, which are often resistant to conventional treatments [8]. Managing NOP remains a significant challenge for healthcare practitioners. Standard pharmacological treatment strategies for NOP include the use of anticonvulsants (*e.g.*, gabapentin, pregabalin), antidepressants (*e.g.*, amitriptyline, duloxetine) and opioids, which are reserved for severe cases [9]. However, these treatments often yield limited success and are associated with dose-limiting side effects, including sedation, dizziness, constipation and nausea, further diminishing patients’ QoL [10, 11, 12, 13]. The chronic nature of NOP necessitates prolonged treatment, exacerbating the burden of side effects and increasing the risk of medication dependency [14]. Consequently, there is growing interest in complementary and integrative therapies that have demonstrated both efficacy and tolerability.

Acupuncture, a fundamental aspect of Traditional Chinese Medicine (TCM), has been recognized as a viable non-pharmacological method for the management of chronic pain, particularly in neuropathic conditions [15, 16]. Acupuncture involves the insertion of very fine, sterile needles into designated anatomical points, called “acupoints”, with the intent of restoring physiological equilibrium and modulating pain pathways [17]. Acupuncture encompasses various techniques, each with distinct mechanisms and applications [18]. Manual acupuncture involves inserting and manipulating needles at specific acupoints, while electroacupuncture (EA) enhances stimulation by applying a mild electric current to the needles. Fire acupuncture utilizes heated needles to promote circulation and alleviate pain, whereas warm needle acupuncture combines needling with moxibustion for enhanced therapeutic effects. Scalp and auricular acupuncture target specific reflex areas on the scalp and ear, respectively, often used for neurological and pain-related conditions. Additionally, abdominal, hand, and foot acupuncture focus on microsystems within the body, addressing both localized and systemic disorders. These diverse acupuncture methods are employed based on TCM principles and modern clinical research to optimize treatment outcomes [19]. Proposed mechanisms of pain relief from acupuncture include the release of endogenous opioids (*e.g.*, endorphins, enkephalins), neurotransmitter modulation (*e.g.*, serotonin, norepinephrine), suppression of pro-inflammatory cytokines (*e.g.*, tumor necrosis factor-alpha (TNF-α), interleukin-1 beta (IL-1β)) and modulation of peripheral and central sensitization. Hence, it stands out as a potential therapeutic agent for the poorly understood pathophysiology of NOP [20, 21, 22, 23]. Emerging evidence suggests significant benefits from dedicated acupuncture for various subtypes of NOP. Studies have documented reductions in pain intensity and improvement in QoL for patients with TN, BMS, TMD and PHN [24, 25]. Nevertheless, the supporting evidence is still limited due to inadequate methodology, small sample sizes and lack of standardization in acupuncture protocols. Other areas requiring further investigation include the long-term efficacy and safety of acupuncture in the management of NOP. The review discusses the progress, controversies and future directions for acupuncture in NOP management, providing a concise overview while addressing current knowledge gaps and potential avenues for future research.

## 2. Mechanisms of acupuncture in neuropathic orofacial pain

NOP, which stems from the interaction between nerve inputs caused by peripheral injury and central sensitization, is marked by persistent pain that often fails to respond to conventional treatments. Due to its diverse mechanisms of action, acupuncture has emerged as a promising therapeutic alternative. In the treatment of NOP, acupuncture predominantly influences both peripheral and central neurochemical processes, neuroplasticity and inflammatory pathways [25, 26]. The following sections outline the primary mechanisms through which acupuncture exerts its effects in managing NOP.

### 2.1 Endogenous opioid release

Acupuncture stimulates the release of endogenous opioids, such as endorphins, enkephalins and dynorphins, which modulate pain perception by binding to opioid receptors in the central and peripheral nervous systems, thereby inhibiting pain signaling and producing analgesic effects [27, 28]. These neuropeptides bind to the μ (mu), δ (delta) and κ (kappa) opioid receptors located in key pain-processing areas such as the periaqueductal gray (PAG), thalamus and dorsal horn of the spinal cord [29]. The activation of these receptors by acupuncture inhibits nociceptive transmission, enhances descending pain inhibition, and reduces pain perception. Repeated acupuncture sessions have been shown to strengthen opioid-mediated pathways and induce neuroplastic changes that persist for long periods [30]. Such adaptations enhance the body’s ability to regulate pain and contribute to long-term analgesic effects. Evidence from animal models and human studies suggests that the release of opioids might also modulate the emotional and psychological aspects of pain, further supporting acupuncture’s holistic benefits in neuropathy pain management [31].

### 2.2 Neurotransmitter regulation

The effect of acupuncture on neurotransmitter dynamics in the CNS is significant, particularly regarding descending inhibitory pathways. It enhances the release of 5-hydroxytryptamine (5-HT) and norepinephrine, both of which are central mediators of descending pain modulation mechanisms [32]. By increasing the levels of these neurotransmitters, acupuncture inhibits endogenous pain signals in the spinal cord and reduces pain perception at various levels of the CNS. Additionally, acupuncture stimulates the activity of gamma-aminobutyric acid (GABA), which helps decrease neuronal excitability and hyperalgesia [33]. By promoting a balanced neurochemical environment, acupuncture not only alleviates pain but also addresses psychological comorbidities such as anxiety and depression, which are common in patients with NOP [34]. These neurochemical effects play a crucial for in breaking the cycle of chronic pain and emotional disturbance, ultimately providing both physical and emotional comfort [35].

### 2.3 Neuroinflammation and immune modulation

Neuroinflammation, resulting from the interactions between neurons, astrocytes and microglia in the CNS, is a hallmark of neuropathic pain. The event of nerve injury triggers inflammatory cascades that lead to the secretion of pro-inflammatory cytokines, such as IL-1β and TNF-α, along with excitatory mediators like glutamate and brain-derived neurotrophic factor (BDNF) [36, 37]. These molecules upregulate nociceptive signaling through the activation of N-methyl-D-aspartate (NMDA) receptors and extracellular signal-regulated kinase (ERK) phosphorylation (pERK), resulting in central sensitization [38]. Acupuncture modulates neuroinflammation by regulating glial cell activation through the Toll-like receptor 4 (TLR4)/nuclear factor-kappa B (NF-κB) signaling pathway. By downregulating pro-inflammatory cytokines (IL-1β, TNF-α, IL-6) and upregulating IL-10, it suppresses excessive immune responses and promotes M2 microglial polarization [39]. TLR4 activation recruits myeloid differentiation primary response 88 (MyD88), triggering NF-κB translocation, which amplifies neuroinflammation [40]. Acupuncture inhibits TLR4/MyD88 signaling, reducing NF-κB-driven cytokine release and preventing pathological glial cross-talk. IL-10 further restrains NF-κB activation, reinforcing an anti-inflammatory state [41]. This modulation mitigates chronic neuroinflammation, supporting its therapeutic role in neuropathic pain (Fig. 1).

**Fig. 1.** Neuroinflammatory Pathways and Cellular Interactions in Nerve Injury-Induced Pain Signaling. Abbreviations: AMPAR: α-amino-3-hydroxy-5-methyl-4-isoxazolepropionic acid receptor; ATP: adenosine triphosphate; BDNF: brain-derived neurotrophic factor; Ca^2^⁺: calcium ion; CX3CR1: C-X3-C motif chemokine receptor 1; ERK: extracellular signal-regulated kinase; FKN: fractalkine; Gln: glutamine; GLS: glutaminase; IL-1β: interleukin-1 beta; IL-1RI: interleukin-1 receptor type I; Na⁺: sodium ion; NMDAR: N-methyl-D-aspartate receptor; NK1: neurokinin-1 receptor; p38: p38 mitogen-activated protein kinase; Perk: phosphorylated ERK; P2X4: purinergic receptor P2X, ligand-gated ion channel 4; SP: substance P; THR: tyrosine kinase receptor; TLR4: toll-like receptor 4; TNF-α: tumor necrosis factor-alpha; TNFR: tumor necrosis factor receptor.

### 2.4 Neuroplasticity and central sensitization

NOP is associated with maladaptive neuroplasticity and central sensitization, both of which are characterized by the increased activity of pain-processing pathways [42]. Functional magnetic resonance imaging (fMRI) studies indicate that acupuncture restores aberrant neural activities in important brain regions such as the anterior cingulate cortex (ACC), insula and somatosensory cortex [43]. This process involves the modulation of neural networks, allowing acupuncture to reduce hypersensitivity to pain and alleviate emotional distress associated with chronic pain [44]. These neuromodulatory effects not only influence pain perception but also contribute to improved emotional regulation and cognitive functioning, highlighting acupuncture’s multifaceted role in treating neuropathic pain [45].

### 2.5 Peripheral modulation

Acupuncture exerts its anti-inflammatory and analgesic effects through the activation of nociceptors and surrounding tissues [42]. It increases local blood flow, enhancing oxygenation and nutrient delivery to the affected area, which accelerates tissue repair and reduces nociceptor sensitization. Additionally, acupuncture promotes the clearance of pro-inflammatory mediators from the extracellular environment [46]. The neuromodulatory action of adenosine further contributes to localized pain relief, owing to its potent anti-inflammatory effects [47]. Adenosine inhibits nociceptive signaling through the A1 receptor, which enables immediate analgesic action and reduces peripheral sensitization [48]. These peripheral mechanisms complement the central effects of acupuncture, offering an integrated framework with both localized and systemic mechanisms contributing to the management of NOP.

## 3. Clinical evidence for acupuncture in neuropathic orofacial pain

Fig. 2 shows some of the major acupuncture points commonly used in treating NOP. These include both local acupoints, such as TE21 (Xia Guan), ST7 (Er Men), ST6 (Jia Che), ST2 (Si Bai), SI18 (Quan Liao), and CV24 (Cheng Jiang), which are located on the face and jaw, and distal points, including LI4 (He Gu) and PC6 (Nei Guan), found on the hand and wrist. Local acupoints mainly target pain and dysfunction within the orofacial region by improving blood circulation, relieving muscle tension and modulating nerve activity. Distal points complement these effects by providing systemic regulation, promoting relaxation and alleviating anxiety symptoms. Together, this combination exemplifies the integrative nature of acupuncture, which combines localized and systemic approaches for effective NOP management.

**Fig. 2.** Commonly utilized acupuncture points for neuropathic orofacial pain management.

### 3.1 Burning mouth syndrome

BMS is a chronic NOP condition characterized by a recurrent burning or dysesthetic sensation in the oral mucosa for over two hours daily and lasting more than three months, without detectable mucosal lesions or abnormal lab results [49]. Despite the absence of detectable physical causes or dental abnormalities, BMS is more prevalent among postmenopausal women and is considered a multifactorial disorder with neuropathic, psychogenic and systemic bases. This complexity, coupled with its uncertain etiology, makes clinical management particularly challenging, as pharmacological interventions often yield inconsistent outcomes. Studies indicate that acupuncture may alleviate BMS symptoms by stimulating specific acupoints, such as SI1 (Shaoze), TE1 (Guanchong), LI4 (Hegu), TW21 (Ermen), ST5 (Daying), ST6 (Jiache) and CV24 (Chengjiang) [49, 50, 51, 52, 53]. These points are selected based on their ability to modulate nociceptive pathways and optimize both local and systemic physiological responses [50, 51, 52, 53]. Mechanistically, acupuncture enhances oral microcirculation, promotes blood flow and restores vascular homeostasis, which links the dysfunction of circulation to BMS pathogenesis [54]. By modulating vasoreactivity, acupuncture may reduce localized neuroinflammation and improve tissue oxygenation. Furthermore, as BMS often involves psychological components such as anxiety and depression, which amplify pain perception and exacerbate the condition, acupuncture appears to improve patients’ emotional well-being [55]. Table 1 provides a concise summary of the pathogenesis and clinical symptoms associated with different subtypes of NOP. Franco *et al*. [56] emphasized that acupuncture combined with auricular therapy offers a dual benefit by addressing both the physical and emotional domains of the disorder. Recent evidence also suggests that acupuncture may influence immunity and hormonal balance, potentially alleviating the neuroinflammatory and neurodegenerative processes underlying BMS [57]. Gremeau-Richard *et al*. [58] conducted a randomized placebo-controlled study on topical clonazepam, demonstrating its effectiveness in reducing BMS symptoms. Heckmann *et al*. [59] performed a double-blind study on systemic clonazepam, which also showed significant symptom relief. Furthermore, Scardina *et al*. [52] reported that acupuncture improved oral microcirculation and alleviated burning symptoms in BMS patients. These studies, along with others, contribute to the growing body of evidence supporting different therapeutic approaches for BMS.

**Table 1.** Summary of most common pathogenesis and clinical symptoms of neuropathic orofacial pain subtypes.

Subtype of NOP	Pathogenesis (Disease Mechanisms)	Symptoms
BMS	Peripheral and central neuropathic changes, including sensory nerve dysfunction. Alterations in central pain processing and small fiber neuropathy. Reduced adrenal and neuroactive steroid levels, leading to neurodegenerative changes. Possible psychological factors (*e.g.*, depression, anxiety). Changes in oral microcirculation and vascular reactivity. Chronic low-grade inflammation and immune dysfunction. Parafunctional habits (*e.g.*, teeth grinding, tongue pressing) exacerbating symptoms. Hormonal changes, particularly in postmenopausal women.	Persistent burning or scalding sensations, often affecting the tongue, lips, hard palate and oral mucosa. Symptoms typically worsen as the day progresses. Altered taste perception (*e.g.*, dysgeusia, metallic taste or bitterness). A feeling of dryness in the mouth despite normal salivary gland function. Oral tingling, numbness or paresthesia. Difficulty speaking, eating or drinking due to discomfort. Psychological distress, including fears of developing cancer. No visible abnormalities observed on clinical examination of the oral mucosa.
TN	Vascular compression of the trigeminal nerve root (*e.g.*, by the superior cerebellar artery) leading to demyelination. Hyperactivity and ectopic discharges of nerve fibers caused by demyelination. Central sensitization and maladaptive neural plasticity in the brainstem and trigeminal nerve nucleus. Potential involvement of neuroinflammation and microvascular changes. Hormonal and age-related factors (commonly observed in individuals over 50 years old).	Sudden, severe, stabbing or electric shock-like pain occurring in the distribution of one or more branches of the trigeminal nerve. The pain is often triggered by mild stimuli, such as speaking, chewing, touching, or exposure to cold air. Pain episodes last from a few seconds to a few minutes, with pain-free intervals in between. Typically unilateral pain affecting the mandibular (V3), maxillary (V2) or ophthalmic (V1) branches of the trigeminal nerve. Psychological symptoms, including anxiety, depression and sleep disturbances, often arise as a result of chronic pain.
PHN	Central and peripheral sensitization: Peripheral nerve injury increases ectopic discharges, leading to heightened pain sensitivity, allodynia and spontaneous pain. Increased expression of voltage-gated sodium and calcium channels: Changes in damaged nociceptors result in heightened excitability. The release of inflammatory mediators (*e.g.*, cytokines, IL-8) affects nociceptive pathways and contributes to the persistence of chronic pain. Loss of inhibitory interneurons and spinal cord alterations: These changes drive central sensitization and prolonged pain states. Sensory nerve dysfunction in affected dermatomes: This dysfunction leads to spontaneous pain and a reduced ability to perceive normal sensations. Abnormal activity in the dorsal root ganglia generates spontaneous neural discharges, perpetuating chronic pain.	Persistent, severe and chronic neuropathic pain: The pain endures for weeks, months or even years following the resolution of herpes zoster rashes. Commonly described as burning, throbbing, stabbing or electric shock-like sensations. Intense pain is often triggered by non-painful stimuli, such as light touch or temperature changes. Physical discomfort, sleep disturbances, anxiety and depression significantly disrupt daily life. Pain restricts mobility and interferes with routine activities. Typically confined to the affected skin area, though in severe cases, it may spread to adjacent regions. Prolonged pain and functional impairment lead to emotional challenges and social isolation.
TMD	TMD arises from a combination of factors, including psychological stress, joint trauma, inflammation, muscle dysfunction and hormonal imbalances. Most associated with masticatory muscle dysfunction, including myofascial pain syndrome and spasm. Involves intra-articular structures, such as disc displacement, osteoarthritis or synovitis within the TMJ. Central and peripheral sensitization amplify pain signals, often linked to neuroinflammatory processes and dysregulated pain modulation pathways. Anxiety, depression and stress exacerbate TMD by influencing muscle tension and pain perception. Local release of inflammatory mediators (*e.g.*, cytokines, prostaglandins) contributes to TMJ pain and dysfunction.	Pain in the TMJ area, often radiating to the face, ear, or temples. Restricted jaw movements, such as difficulty in opening the mouth or chewing. Audible joint noises (*e.g.*, clicking, popping, or grinding sounds) during jaw movements. Joint stiffness and tenderness in the masticatory muscles, exacerbated by jaw movement. Headaches, particularly tension-type headaches, often co-occurring with neck and shoulder discomfort. Tinnitus or aural fullness, sometimes accompanied by mild hearing impairment.

*BMS: burning mouth syndrome; PHN: postherpetic neuralgia; TMJ: temporomandibular joint; TMD: temporomandibular disorders; TN: trigeminal neuralgia. NOP: neuropathic orofacial pain; IL: interleukin.*

### 3.2 Trigeminal neuralgia

TN presents substantial clinical management challenges owing to its insidious onset and limitations of conventional pharmacotherapy. Although carbamazepine maintains its status as first-line treatment, its therapeutic utility is frequently constrained by dose-dependent adverse effects including dizziness, fatigue and cognitive impairment, which collectively compromise patients’ QoL [60]. Emerging evidence positions acupuncture as a promising therapeutic alternative, demonstrating statistically significant pain reduction and enhanced treatment response rates compared to conventional approaches.

A comprehensive network meta-analysis by Yin *et al*. [61], encompassing 58 randomized controlled trials (RCTs) with >4000 participants, revealed that combined EA and manual acupuncture protocols outperformed carbamazepine monotherapy in both pain intensity reduction (measured by visual analogue scale improvements) and achieving clinically meaningful response rates (defined as ≥50% pain relief). This therapeutic advantage was particularly evident when compared to sham acupuncture controls. Complementary findings by Gao *et al*. [60] further substantiate acupuncture’s multidimensional benefits, demonstrating concurrent improvements in cognitive function and QoL metrics alongside pain alleviation. The robustness of these conclusions is reinforced by multicenter studies from Edwards *et al*. [62] and Hu *et al*. [63], which confirmed consistent efficacy across varied acupuncture modalities including EA, traditional manual needling, and fire acupuncture techniques. Acupuncture also performed well in terms of safety, with few documented side effects across the studies [64, 65]. Yin *et al*. [61] specifically noted an absence of serious adverse events across analyzed trials, positioning acupuncture as a well-tolerated option for medication-intolerant patients. Clinical application in TN typically involves strategic stimulation of trigeminal nerve-associated acupoints: ST2 (Sibai), ST4 (Dicang), ST6 (Jiache) and ST7 (Xiaguan) for local neuromodulation, combined with distal points including LI4 (Hegu), GB20 (Fengchi) and DU26 (Renzhong) for systemic regulation [60, 61, 66, 67, 68]. This dual-target approach concurrently addresses peripheral nociceptive pathways and central pain processing mechanisms, potentially explaining its sustained therapeutic effects.

### 3.3 Temporomandibular disorders

TMD encompasses a spectrum of conditions affecting the temporomandibular joint (TMJ), masticatory muscles, and associated structures, often presenting with chronic orofacial pain, myofascial discomfort, and joint dysfunction [69]. Symptoms such as restricted jaw mobility, clicking or popping sounds, and persistent muscular pain significantly impair patients’ QoL [70]. While conventional treatments, including physical therapy, occlusal splints and pharmacological interventions, are widely utilized, their efficacy varies, and adverse effects or incomplete symptom relief often limit their applicability. This has driven growing interest in adjunctive therapies like acupuncture and EA, which have demonstrated potential in symptom relief, muscle relaxation and functional improvement of the TMJ [71]. Acupuncture and EA have been extensively studied for their capacity to reduce pain, regulate neuromuscular activity, and address the inflammatory mechanisms underlying TMD. Key acupoints used in treatment include SI18 (Quanliao), TW21 (Ermen), LI4 (Hegu), ST6 (Jiache), ST7 (Xiaguan), GV24 (Shenting), GV20 (Baihui), GV21 (Qianding), GV22 (Xinhui), ST36 (Zusanli), LI3 (Sanjian) and LI11 (Quchi) [72, 73, 74, 75, 76, 77, 78, 79, 80]. These points are chosen for their ability to alleviate pain, improve joint function and regulate neuromuscular activity. These acupoints are specifically selected for their roles in modulating pain pathways, improving joint mobility, and promoting neuromuscular coordination. Studies consistently report significant reductions in pain intensity and enhancements of jaw function when acupuncture or EA is employed, often outperforming sham acupuncture or standard care. For example, Liu *et al*. [80] demonstrated substantial reductions in pain scores and improvements in jaw mobility, with effects persisting for weeks post-treatment. Similarly, Sung *et al*. [76] found that EA combined with ultrashort wave therapy provided superior therapeutic outcomes, indicating a synergistic effect when acupuncture is integrated with other modalities.

The mechanisms underlying acupuncture’s efficacy in TMD are multifaceted, involving both peripheral and central processes. Acupuncture stimulates the release of endogenous opioids, such as β-endorphins, and modulates serotonergic and dopaminergic pathways, both of which are essential in pain regulation and perception [81]. Furthermore, acupuncture has been shown to attenuate inflammatory responses by downregulating cytokines such as TNF-α and IL-6, both of which contribute to TMD symptom severity [42]. EA amplifies these benefits by delivering electrical stimulation to acupoints, further improving nerve conduction, reducing muscular hyperactivity and facilitating localized blood flow [82]. Acupuncture’s impact extends beyond the physical symptoms of TMD, addressing the psychological dimensions of the disorder. Anxiety, depression and stress, which are prevalent among TMD patients, often exacerbate pain perception and complicate treatment outcomes. Acupuncture has been shown to modulate the hypothalamic–pituitary–adrenal (HPA) axis, reducing cortisol levels and promoting emotional regulation [83]. Jung *et al*. [77] observed that acupuncture not only alleviated muscle tension but also improved emotional function, highlighting its role in mitigating the both physical and psychological burden of TMD. These effects underscore the holistic potential of acupuncture in addressing both the somatic and psychological dimensions of the disorder. However, discrepancies in the reported efficacy of acupuncture persist. Wu *et al*. [84] and Laurence *et al*. [78] found mixed results, with limited evidence supporting acupuncture’s superiority over sham treatments in certain contexts. These inconsistencies can be attributed to methodological variability, including differences in diagnostic frameworks, acupoint selection, treatment protocols and study designs. Such heterogeneity underscores the need for rigorously designed, standardized studies to establish clearer evidence regarding acupuncture’s therapeutic role in TMD. Future research should prioritize randomized, sham-controlled trials with well-defined diagnostic criteria and standardized acupuncture protocols to ensure reliability and reproducibility. These findings collectively suggest that acupuncture and EA offer a multifaceted approach to TMD management, targeting not only localized pain and inflammation but also systemic and psychological factors.

### 3.4 Postherpetic neuralgia

PHN is a challenging form of chronic neuropathic pain that persists even after the remedy of herpes zoster infection [85]. It is often characterized by severe, localized pain along affected dermatomes. Treatment remains complex due to the intricate interplay of peripheral and central sensitization mechanisms, which allow symptoms to persist and intensify [86]. Conventional treatments encompass all the usual approaches, including anticonvulsants and topical treatments; unfortunately, they usually offer only partial relief, with scant benefits due to the side effects experienced and the continued persistence of some symptoms [9]. Acupuncture is thought to reduce PHN symptoms via a multifaceted modulation of neural and inflammatory pathways: its mechanism of action includes inhibiting peripheral sensitization by reducing the release of proinflammatory cytokines such as IL-6 and TNF-α, which are among the most critical mediators of nociceptive sensitization [42, 46]. Acupuncture also regulates the transient receptor potential vanilloid 1 (TRPV1) activity, which may help diminish nociceptive sensitivity in the dermatomes [87]. From a central perspective, acupuncture reduces dorsal horn hyperexcitability by downregulating NMDA receptor activation and enhancing opioid receptor-mediated analgesic pathways [20]. These effects are often exaggerated by releasing endogenous opioids (endorphins and enkephalins) and modulating GABAergic and serotonergic transmission, all of which contribute to altered pain perception [88]. By modulating sympathetic and parasympathetic responses, acupuncture harmonizes different functions of the autonomic nervous system and hence reduces the stress-induced exacerbation of pain [89]. Acupuncture for PHN involves the careful selection of acupoints to address both localized and systemic pathophysiological processes. Commonly used points include Ashi points (non-fixed points that are selected based on tenderness or pain), Jiaji (EX-B2), GB34 (Yanglingquan), LI4 (Hegu), ST36 (Zusanli), GB21 (Jianjing), LR3 (Taichong), SP6 (Sanyinjiao), BL15 (Xinshu), and PC6 (Neiguan) [64, 65, 66, 67, 68, 69]. Concisely, ashi points are chosen for diminishing localized inflammation and pain at affected dermatomes, while Jiaji (EX-B2), located near the spinal column, exerts its effects through modulation of central sensitization and autonomic balance [90]. Other systemic points, like ST36 (Zusanli) and LI4 (Hegu), are selected for their ability to enhance microcirculation, reduce systemic inflammation and promote neural repair, reflecting the integrative approach to acupuncture in PHN management [91].

Several acupuncture techniques have shown therapeutic efficacy in PHN, including EA, manual acupuncture, fire needling, moxibustion (a therapy that involves burning dried mugwort near the skin to stimulate acupoints), bloodletting-cupping (a method combining controlled bleeding and cupping to promote circulation and remove stagnation) and acupoint injection (the injection of medicinal substances into acupuncture points to enhance therapeutic effects) [65]. For instance, Pei *et al*. [92] reported significant pain relief and improved sleep quality with low-frequency EA [92]. Similarly, Wang *et al*. [93] found that fire needling demonstrated superior curative effects compared to other techniques, as it combines thermal stimulation with traditional needling methods [93]. Ashi points, treated by bloodletting cupping, have shown substantial pain relief although the risks of mild adverse effects (*e.g.*, dizziness or bleeding) do increase a little [65]. Both moxibustion and acupoint injection promoted circulation and reduced inflammation through heat stimulation. Additionally, acupoint injection integrated acupuncture with pharmacological agents, such as vitamin B12, to enhance therapeutic effects [65, 94]. Improvements in pain scores and QoL continue to support acupuncture as a potentially safe and effective strategy for PHN. However, challenges remain in standardizing acupuncture protocols and confirming its long-term efficacy. As acupuncture becomes more integrated with conventional therapies, its therapeutic potential is expected to grow, providing a more comprehensive approach to managing this debilitating condition.

### 3.5 Clinical applications

Acupuncture has been widely studied for its effects on neuromodulation, pain regulation and neurovascular function, demonstrating clinical relevance in the management of NOP. The selection of acupoints is guided by anatomical and neurophysiological considerations, with research supporting their role in pain modulation and neural activity regulation. For instance, LI4 (Hegu) has been shown to influence pain perception by modulating neurotransmitter release and improving microcirculation, contributing to its effectiveness in conditions such as facial paralysis, TN and TMD [66, 95, 96]. Clinical studies suggest that LI4 stimulation enhances endogenous pain control mechanisms, reducing hyperalgesia and central sensitization [20]. Additionally, ST36 (Zusanli) and LI11 (Quchi) are frequently utilized due to their systemic effects on immune function, neural excitability and inflammatory response, making them valuable in broader neuropathic conditions [23, 97]. Acupoint selection is often tailored to specific conditions, aligning with pathophysiological mechanisms. For example, SI18 (Quan Liao) and TE21 (Er Men) are commonly employed in TMD treatment, aiding in neurovascular regulation and muscle function restoration [98]. Similarly, EX-HN5 (Taiyang) and DU26 (Ren Zhong) are frequently utilized in TN management, targeting cranial nerve dysfunction and neuropathic pain pathways [99]. Moreover, scalp acupuncture points, such as GV20 (Baihui) and GV24 (Shen Ting), have been investigated for their role in modulating cortical activity and cranial nerve function, making them relevant in neurological conditions involving central pain processing and neurovascular dysregulation [100]. These findings underscore the importance of precision in acupoint selection, ensuring that therapeutic interventions align with the underlying neurophysiological mechanisms of pain and dysfunction.

By integrating acupuncture with contemporary pain management strategies, clinicians can enhance therapeutic outcomes in NOP and associated neuropathic syndromes. The neuromodulatory effects of acupuncture, supported by functional anatomy and neurophysiological mechanisms, reinforce its role as a scientifically supported adjunct therapy in neuropathic pain management. While acupuncture shows promise as a non-pharmacological treatment, several barriers hinder its clinical implementation. The lack of standardized protocols leads to variability in practice, while inconsistent training requirements affect treatment quality. Patient adherence can also be challenging, as multiple sessions are often required for effectiveness. Additionally, limited insurance coverage and regulatory inconsistencies restrict accessibility. Addressing these issues through standardized guidelines, improved practitioner training, and policy support is essential for optimizing acupuncture’s clinical integration.

### 3.6 Limitations and future directions

Despite promising findings, the current body of evidence on the use of acupuncture for NOP remains limited primarily due to significant methodological shortcomings. Key issues include inconsistencies in acupoint selection, treatment frequency, session duration and study design, all of which compromise the reproducibility and comparability of findings. Additionally, short follow-up periods raise concerns about the reliability and broader applicability of reported outcomes. Furthermore, the lack of standardized protocols tailored to the various subtypes of NOP further complicates the development of precise clinical guidelines. Interpreting data from acupuncture trials is further challenged by difficulties in controlling placebo effects and the insufficient exploration of its long-term therapeutic benefits. Moreover, the limited research on combining acupuncture with other non-invasive therapies highlights a significant gap in the existing evidence, underscoring the need for future exploration of such integrative approaches [94, 101, 102]. To address these issues, future studies must prioritize the adaptation of standardized protocols and extend their duration to evaluate both the immediate and lasting effects of acupuncture. Advancing the field will require detailed mechanistic studies using tools such as functional MRI to investigate how acupuncture influences processes like central sensitization, neuroinflammation and neural plasticity. Comparative trials are also essential to assess the combined efficacy of acupuncture with established pharmacological and non-pharmacological treatments. The lack of comprehensive research in these domains underscores an unexploited opportunity to optimize acupuncture’s role in managing chronic pain conditions. By addressing these research priorities, the field can progress toward developing evidence-based guidelines, unlocking the full potential of acupuncture as a treatment for NOP.

## 4. Conclusions

Existing clinical studies highlight the therapeutic potential of acupuncture in the treatment of NOP demonstrating its ability to alleviate pain and enhance QoL. As an integrative therapy, acupuncture offers a promising approach, however, several significant challenges remain, including a limited understanding of its precise mechanisms and the absence of standardized protocols for acupoint selection, techniques and treatment regimens. Future research should prioritize a multidisciplinary approach, integrating robust experimental designs to deepen insights into the neurophysiological mechanisms underpinning acupuncture’s effects. Beyond these investigative efforts, greater emphasis must be placed on developing standardized protocols for acupoint selection and procedural techniques to ensure consistency and improve treatment outcomes. Addressing these critical gaps would establish a stronger foundation for acupuncture as a reliable and optimized therapeutic option, offering meaningful relief to individuals suffering from NOP.
